# Effects of Interphase and Interpulse Delays on Tissue Impedance and Pulsed Field Ablation

**DOI:** 10.1007/s10439-025-03757-4

**Published:** 2025-05-16

**Authors:** Edward J. Jacobs, Pedro P. Santos, Rafael V. Davalos

**Affiliations:** 1 Wallace H. Coulter Department of Biomedical Engineering, Georgia Tech – Emory University, U.A. Whitaker Building, 313 Ferst Drive, Suite 2101, Atlanta, GA USA; 2 School of Electrical Engineering, Georgia Tech, Atlanta, GA USA

**Keywords:** Pulsed field ablation, Impedance, High-frequency irreversible electroporation, Delays, Cardiac

## Abstract

**Purpose:**

High-frequency irreversible electroporation (H-FIRE) is a pulsed field ablation (PFA) technique that employs a series of high-voltage, microseconds-long positive and negative pulses, separated by interphase (d1) and interpulse (d2) delays to non-thermally ablate tissue. Previous experimental and computational data suggest an impact of delays on nerve excitation and electrochemical effects. However, the impact of delays on PFA outcomes, such as change in resistance and ablation generation, has only recently started to be elucidated.

**Methods:**

While recording the applied voltage and currents, we delivered a series of increasing voltages, termed voltage ramps, into tuber and cardiac tissues using both needle electrode pairs and flat plate electrodes. Tissues were stained for metabolic activity to measure irreversible electroporation areas following treatment.

**Results:**

Our findings support previous *in vitro* data that delays do not significantly affect ablation areas. While there were significant differences in applied current, resistance, and conductivity between different pulse widths at sub-electroporation electric fields, we found no significant differences after inducing electroporation between different delays and pulse widths. Consequently, since delays do not affect ablation areas or local conductivity, the data suggests that delays should not affect the electric field threshold or Joule heating within the tissue.

**Conclusion:**

The findings presented here provide critical insights into electroporation-dependent tissue conductivity changes from H-FIRE with implications for improving H-FIRE parameterization and computational models for treatment planning in cancer and cardiac pulsed field ablation.

**Supplementary Information:**

The online version contains supplementary material available at 10.1007/s10439-025-03757-4.

## Introduction

Minimally invasive ablation modalities are integral in modern surgical intervention of benign and malignant neoplasms, where it is necessary to remove undesirable tissue while minimally affecting the surrounding parenchyma and critical structures. Thermal ablation (e.g., histotripsy, radiofrequency ablation, and cryoablation) is the mainstay for many soft tissue neoplasms [1, 2]. However, from a technical perspective, tumor size and location (adjacent to critical structures) are the most common factors limiting thermal ablation use, and there is a significant risk for non-tumor tissue loss, damage to critical structures, and subsequent complications [3–6].

Irreversible electroporation (IRE) is a technique developed by Davalos et al. [7] and used clinically to treat unresectable and aggressive tumors of the prostate [8–10], kidney [11–13], liver [14–16], and pancreas [17–19]. IRE typically employs high-voltage (1000−3500 V), microseconds-long (70−100 µs) monopolar pulses applied between minimally invasive electrodes inserted within or adjacent to the tumor [20]. The generated electric field causes an ionic current within the tissue that induces an electric potential on the plasma membrane of cells within the tissue. Once the induced transmembrane potential reaches a critical value (~ 0.2–1 V) [21, 22], the membrane permeabilizes through the formation of nanoscale defects (electroporation) [23, 24]. If persistent, the cells within a critical electric field threshold (EFT) die through loss of homeostasis [25]. Through careful treatment planning, large ablation volumes (> 50 cm^3^) can be achieved in soft tissue using IRE [26, 27] without significant heating of surrounding tissue or structures [96]. The nonthermal mechanism of IRE allows for the treatment of masses near biliary structures, bowel tissue [28], ducts [29], mature blood vessels [30], and nerves [31]. Thus, IRE is becoming an increasingly important approach to treat patients with primary or metastatic disease in locations where surgical resection and thermal ablation methods are contraindicated [32–35].

**Fig. 1:**
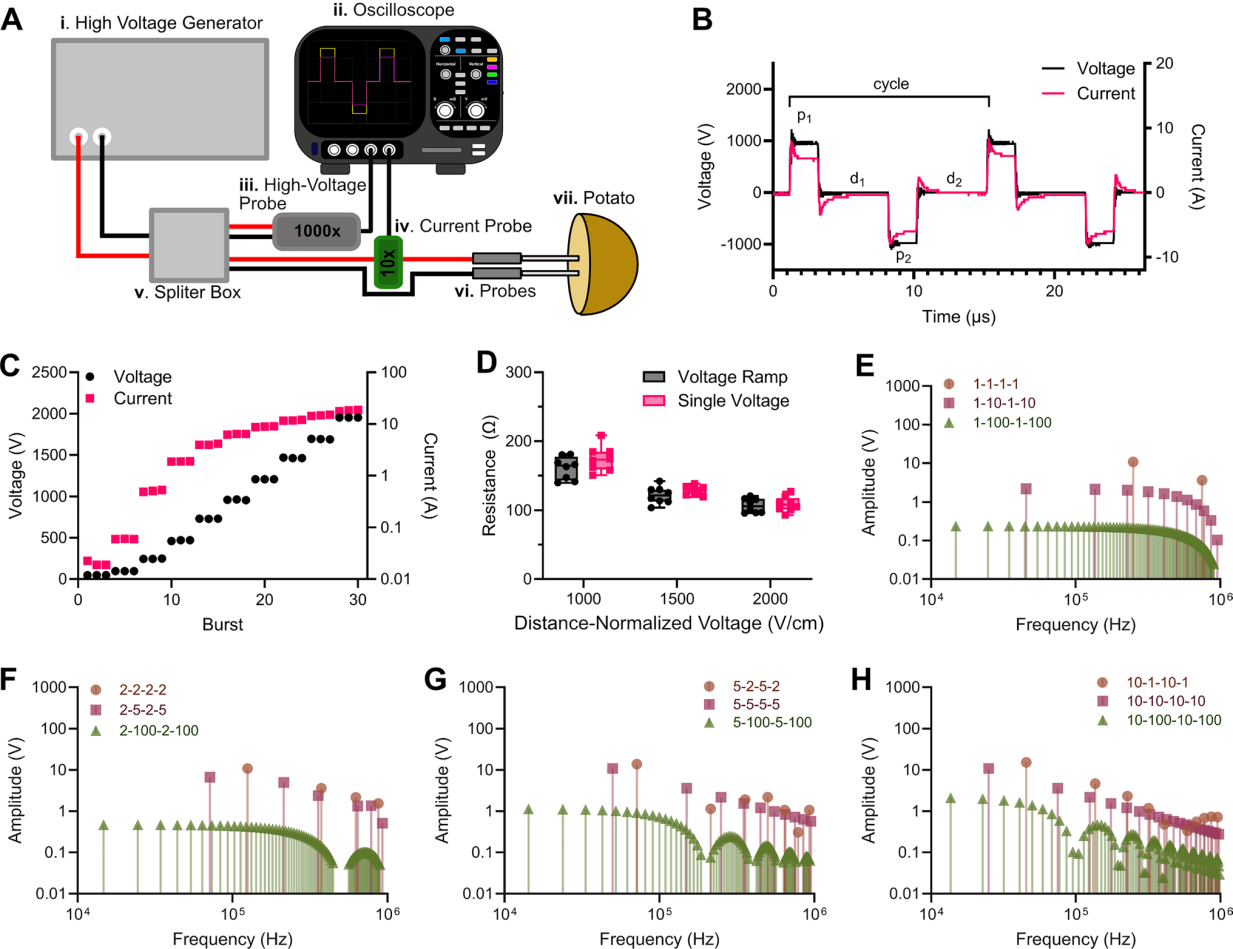
**A** Experimental Setup. The voltage and current are generated by a (i) custom high-voltage generator and are monitored using an (ii) oscilloscope attached to a (iii) 1000x attenuated high-voltage probe and (iv) 10x attenuated current probe. A (v) high-voltage splitter box directs the current either to the oscilloscope or to the (vi) treated tissue (i.e., potato or heart) with two monopolar NanoKnife^®^ probes inserted. **B** Voltage and current for 2 cycles of a representative 2–5-2–5 [positive pulse—interphase delay—negative pulse—interpulse delay], recorded on the oscilloscope. **C** Representative voltage and current data for 1 voltage ramp. After inserting the probe, the voltage ramp applies a series of 3 bursts, increasing the voltage each ramp. **D** Resistance measurements for the final applied voltage within a voltage ramp or for that voltage alone, with no preceding ramps. Impedance distribution of **E** a 1 µs, **F** a 2 µs, **G** a 5 µs, and **H** a 10 µs pulse width H-FIRE waveform with delays varying from 1 µs to 100 µs. (Panel D: *n* = 8)

However, the [relatively] long pulses (70−100 µs) employed with IRE require systemic, intraoperative neuromuscular blockades to reduce involuntary muscle contractions that can lead to electrode displacement during treatment [36–38]. Further, pulse delivery must be synchronized with the cardiac cycle to avoid potential cardiac arrhythmia [39]. To overcome these limitations, Arena et al. developed high-frequency IRE (H-FIRE) [40], which utilizes a series of short (0.5–10 µs), bipolar pulses. The H-FIRE burst is constructed of a positive pulse, interphase delay (d_1_), negative pulse, and interpulse delay (d_2_), repeated for a number of cycles to achieve a total on-time comparable with conventional IRE (Fig. 1B).

Catheter-based H-FIRE has had great success recently for treating cardiac arrhythmias with similar efficacies, but significantly lower complication rates, compared to thermal ablation [41–43]. However, due to the unique and multifaceted outcomes achievable by adjusting the H-FIRE waveforms, no standard or optimized H-FIRE protocol has been established to date, and the impact of H-FIRE parameterization on clinical outcomes (i.e., ablation volume, thermal heating, nerve excitation, and type of cell death) has only recently started to be resolved. Pulse width is the most studied parameter and is well known to have a positive correlation with ablation size and a negative correlation with the lethal electric field threshold [44–46]. The rate that bursts are applied is also shown to moderately influence lethal thresholds, with higher burst rates lowering the lethal threshold [47]. Increasing the number of bursts also significantly increases ablation size [44, 48]. Computational models of neuromuscular excitation have suggested that shorter interphase delays and longer interpulse delays theoretically decrease neuromuscular excitation [49]. Following, shorter interphase delays were experimentally demonstrated to decrease neuromuscular excitation and pain during H-FIRE delivery [50]. Further, a decreased interval between bursts and an increased number of cycles within each burst is also implicated to increase neuromuscular excitation [48].

Here, we interrogate the impact that H-FIRE parameterization has on experimental outcomes. We utilized a potato tissue model and fresh ex vivo porcine cardiac tissue to evaluate the impact of pulse width, interphase delay, and interpulse delay on tissue resistances, conductivities, and pulsed field ablation.

## Methods

### Potato Experimental Set Up

*Solanum* Tuberosum (Russet potatoes, Publix) were used within 2 days of purchase, and all experiments were conducted at room temperature (23 °C, measured). Potatoes were cut in half-perpendicular to the longest axis. Two 1-mm diameter NanoKnife^®^ monopolar probes (AngioDynamics Inc.) were used to deliver electrical pulses through the tissue. A custom 3D-printed spacer was used to maintain a 1.0 cm or 1.5 cm center-to-center spacing between the probes. Probe tips were sanded to remove the sharp tip, and the electrode exposure was measured to be 1.0 cm for all treatments. Both probes were simultaneously inserted into the potato so that the midline of the electrode exposures were 2.5 cm deep into the potato.

### Cardiac Experimental Set Up

Whole porcine hearts were obtained from female Yorkshire-Landrace pigs (1–2 years old, estimated 200 kg at euthanasia) at the University of Georgia Meat Science Technology Center. As transit of tissue on ice can affect viability and potentially electroporation effects, all experiments for a given heart were performed at the meat center within 20 minutes of death to maintain tissue viability and body temperature. Hearts were removed within 10 minutes of death and all experiments were performed within 10 minutes of receiving the heart. Hearts were not perfused as to apply electroporation as soon as possible once removed from the body. Temperature within the ventricle of the heart was measured to be stable at body temperature (~ 37–40 °C, measured) throughout the experiments.

Due to the thickness of the porcine ventricle (~ 15−20 mm, measured), we could not utilize the NanoKnife^®^ monopolar probes. Instead, small stainless steel acupuncture needle electrodes were used for the experiments (Fig. 6A). The electrode spacing was 1 cm. The electrode diameter was 0.45 mm, and the electrode exposure was 4 mm for both geometries. The electrodes were inserted into the left ventricle from the outside of the heart so that the midline of the electrode exposure was 8 mm deep.

### Application of Pulsed Electric Fields

Applied pulsed electric fields were generated using a custom high-voltage pulse generator (OmniPorator, VITAVE) according to the schematic in Fig. 1A. Applied voltages and currents were monitored using a WaveSurfer 4024HD 5 GHz oscilloscope (Teledyne LeCroy) equipped with a 1000 × attenuated high-voltage probe (DPB5700, Siglent) and a 10 × attenuated current probe (3972, Pearson Electronics). A fixed sampling period of 2 ns was maintained between each waveform to ensure that each positive and negative pulse contained at least 500 data points (Fig. 1B).

Without moving the inserted probe, the generator was set to deliver a series of increasing voltages (Fig. 1C), termed a voltage ramp (VR). The voltage ramp consisted of 3 bursts, repeated in series and in ascending magnitude. To normalize between different probe spacings (1.0 cm and 1.5 cm), we calculated the voltage to achieve distance-normalized voltage (i.e, applied electric field) of 50, 100, 250, 500, 750, 1000, 1250, 1500, 1750, and 2000 V/cm. Due to the increased current required for the 1.5 cm spacing compared to the 1.0 cm spacing, the maximum ramp applied was 1500 V/cm. For potatoes, after VRs were successfully delivered, a treatment of 100 bursts was delivered at the same location without removing the probes. Since the needle electrodes had a smaller spacing and less applied current, VRs up to 2500 V/cm were applied for cardiac tissue. However, due to the lower limit of deliverable voltage on the generator (50 V), the lowest applied distance-normalized voltage was 100 V/cm.

### Parallel Flat Plate Potato Experiments

To deliver a uniform electric field, we utilized parallel flat plate electrodes attached to a caliper (Fig. 5A). We cut the potato to produce ~ 6 mm slices. We then used a 6 mm diameter biopsy punch to generate cylinders of tissue. To account for slight variations in thicknesses between samples, we measured the thickness of the potato cylinder with the calipers (3415 FS, Fischer Scientific). We then calculated the voltage needed to deliver uniform electric fields of 100, 250, 500, 750, 1000, 1250, 1500, 1750, and 2000 V/cm through the tissue cylinder. Due to the lower limit of deliverable voltage on the generator, the lowest voltage ramp was set to 60 V (i.e., 100 V/cm).

### Potato Ablation Area Measurements

After treatment using the 2 monopolar probes, a line was drawn on the outside of the potato to designate where the midline (2.5 cm deep) of the electrode insertion was. The potatoes were maintained for 24 hours at room temperature to allow for ablations to develop. At 24 h, we cut the potatoes through the midline of the electrode using the line as a guide. Then, a mandolin was used to produce a flat 3 mm cross-sectional slice for consistent imaging distances. We then imaged the potato on a grid cutting mat using a digital camera (Fig. 4A). A custom holder was used to maintain 16.5 cm from the surface for every image. The images were then imported into ImageJ (National Institutes of Health) for analysis. The dark melanin region for each potato was measured using the oval selection tool. The pixel value for the length of the 1 cm grid spacing was used to convert the measured pixel areas from ImageJ into centimeters squared.

### Voltage and Current Data Processing

Voltage and current data were initially processed through a digital first-order low-pass filter with a time constant ($\tau$) equal to one-third of the applied waveform’s pulse width. This produced an objective method to where we could quantify the voltage or current magnitudes and was useful for short pulse widths (< 2 µs) where capacitance effects from charging the tissue impacts the value measured at the pulse plateau. The maximum value of the processed data for each pulse was then extracted, and the median of these maximum values within a burst was used to calculate the voltage and current for that burst. Since we are measuring the flat portion of the pulses, we estimate the resistance under a quasistatic condition with Ohm’s law, using the median of the voltage values for each pulse and the median of the current values for each pulse as previously done [51–54]. The final voltage, current, and resistance values for a specific ramp were calculated as the simple average of the three bursts.

### Potato Conductivity Measurements

Potato tissue conductivities for the parallel flat plate potato treatments were calculated from the measured resistance using the shape factor for a prism [55]. The conductivity is calculated using:

$$\sigma =\frac{l}{A\cdot {R}_{B}}$$

where $\sigma$ is the tissue conductivity, $l$ is the thickness of the sample, $A$ is the circular area of the cylinder, and ${R}_{B}$ is the measured resistance of the burst.

### Statistical Analysis

*A priori* sample size calculations were performed using G*Power 3.1 (Universität Düsseldorf) to achieve a power of *β* > 0.8 at significance level of *α* = 0.05 [56]. Prism version 10 (GraphPad Software) was employed for all statistical analysis with significance level of *α* = 0.05. Comparisons between two groups were calculated with an unpaired two-tailed Welch’s T-test, and comparisons for 1-factor between multiple groups were calculated with a one-way ANOVA with Tukey’s post hoc test.

## Results

### The Lower Applied Voltages in the Voltage Ramp do not Affect the Current or Resistance Measurements of Subsequent Higher Voltages

To accurately characterize the minute differences in tissue response to electric pulses, it is necessary to compare the response of the same tissue to different electric fields [57]. However, it is not currently known if the application of lower electric fields impacts the resistances read during treatment using higher electric fields within tissue, specifically for voltage ramps. Therefore, we first quantified potato tissue resistance after applying either a voltage ramp up to a target-applied distance-normalized voltage (i.e., 1000 V/cm, 1500 V/cm, or 2000 V/cm) or only the ramp at that target voltage. We found no significant difference in resistance measurements between the voltage ramp and standalone ramp condition for every target-applied distance-normalized voltage group (Fig. 1D).

### The Applied Currents and Measured Resistances During H-FIRE Pulses are Not Affected by Interphase and Interpulse Delays.

**Fig. 2:**
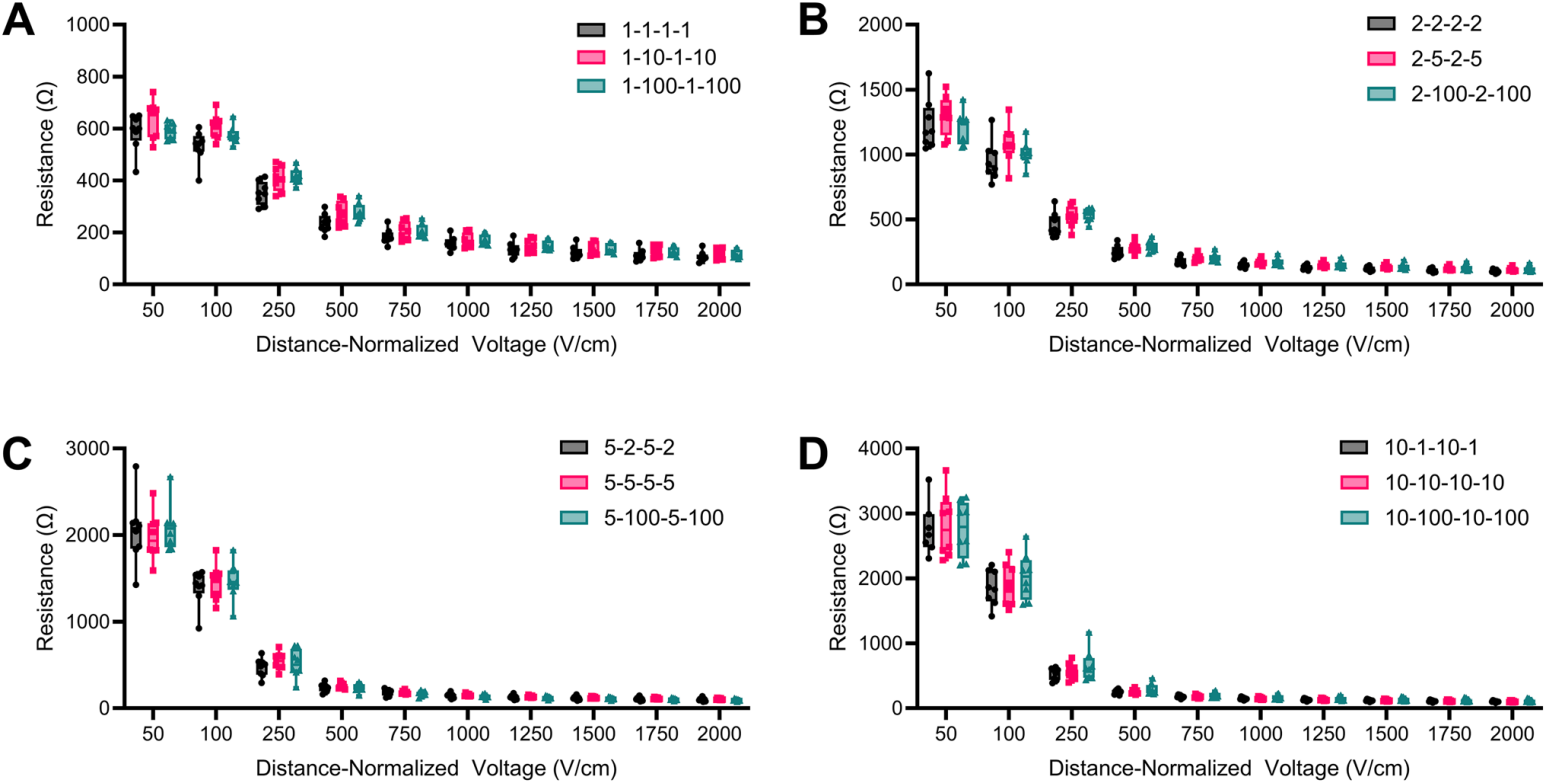
There is not a significant difference in tissue resistance when varying delays. Resistance was measured using 2 monopolar probes with a 1.0 cm center-to-center spacing. Resistance measurement from 50 V/cm to 2000 V/cm for **A** 1 µs, **B** 2 µs, **C** 5 µs, and **D** 10 µs pulse width H-FIRE waveforms with delays varying from 1 µs to 100 µs. (Panels A–D: *n* = 8)

Previous papers have used the impedance spectrum of the waveform to estimate tissue conductivity at specific characteristic frequencies [49, 52]. Here, we constructed 12 H-FIRE waveforms for comparison, using 4 pulse widths (1 µs, 2 µs, 5 µs, and 10 µs) and 3 delays, ranging from 1 µs to 100 µs. Each waveform has a unique impedance spectrum (Fig. 1E–H); however, we found that for each pulse width, there was no significant difference in measured resistance (Fig. 2A–D) or applied current (Supplemental Fig. 1A–D) when varying the delays from 1 µs to 100 µs in potatoes.

**Fig. 3:**
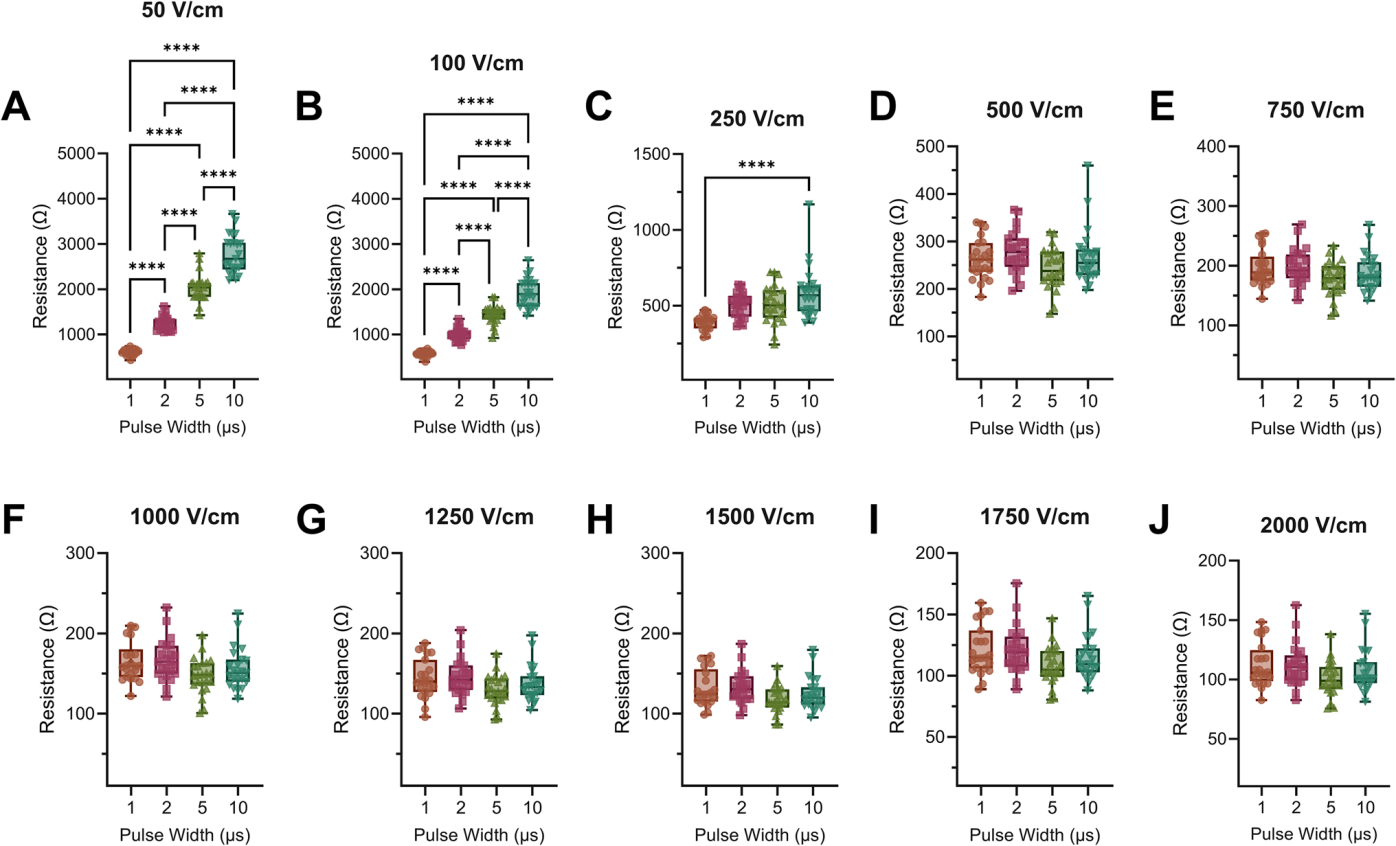
There is only a significant difference in tuber tissue resistance at low applied distance-normalized voltages. **A** At sub-electroporation thresholds, there is a significant difference for resistances between pulse widths, with higher pulse widths having higher resistances. **B** As the tissue begins to electroporate, there is still a significant difference between different pulse widths, but the overall resistance significantly drops **C** at 250 V/cm, there is only a significant difference in resistance between the 1 µs and 10 µs pulse widths. **D**–**J** At high applied distance-normalized voltages, there is not a significant difference in resistance between pulse widths. (Panels A–J: *n* = 24; *p < 0.05, **p < 0.01, ***p < 0.001, ****p < 0.0001)

To investigate how pulse width affects applied current and resistance in tissue, we then clustered each waveform by pulse width. For 50 V/cm, there was a significant difference in measured resistance (Fig. 3A) and current (Supplemental Fig. 2A) between each pulse width. For 100 V/cm, there was a significant difference in resistance between every group (Supplemental Fig. 2B) and current, except between 5 µs and 10 µs (Supplemental Fig. 2B). For 250 V/cm, there was only a significant difference in resistance between 1 µs and 10 µs (Fig. 3C), and there was only a significant difference in current between 1 µs and 5 µs and between 1 µs and 10 µs. For applied distance-normalized voltages from 500 V/cm to 2000 V/cm, there was no significant difference for current or resistance between any pulse widths.

To determine whether the trends persisted in potatoes at a different geometry, we increased the center-to-center spacing between the electrodes from 1.0 cm to 1.5 cm. We again demonstrated that there is not a significant difference when varying delays (Supplemental Fig. 3).

**Fig. 4:**
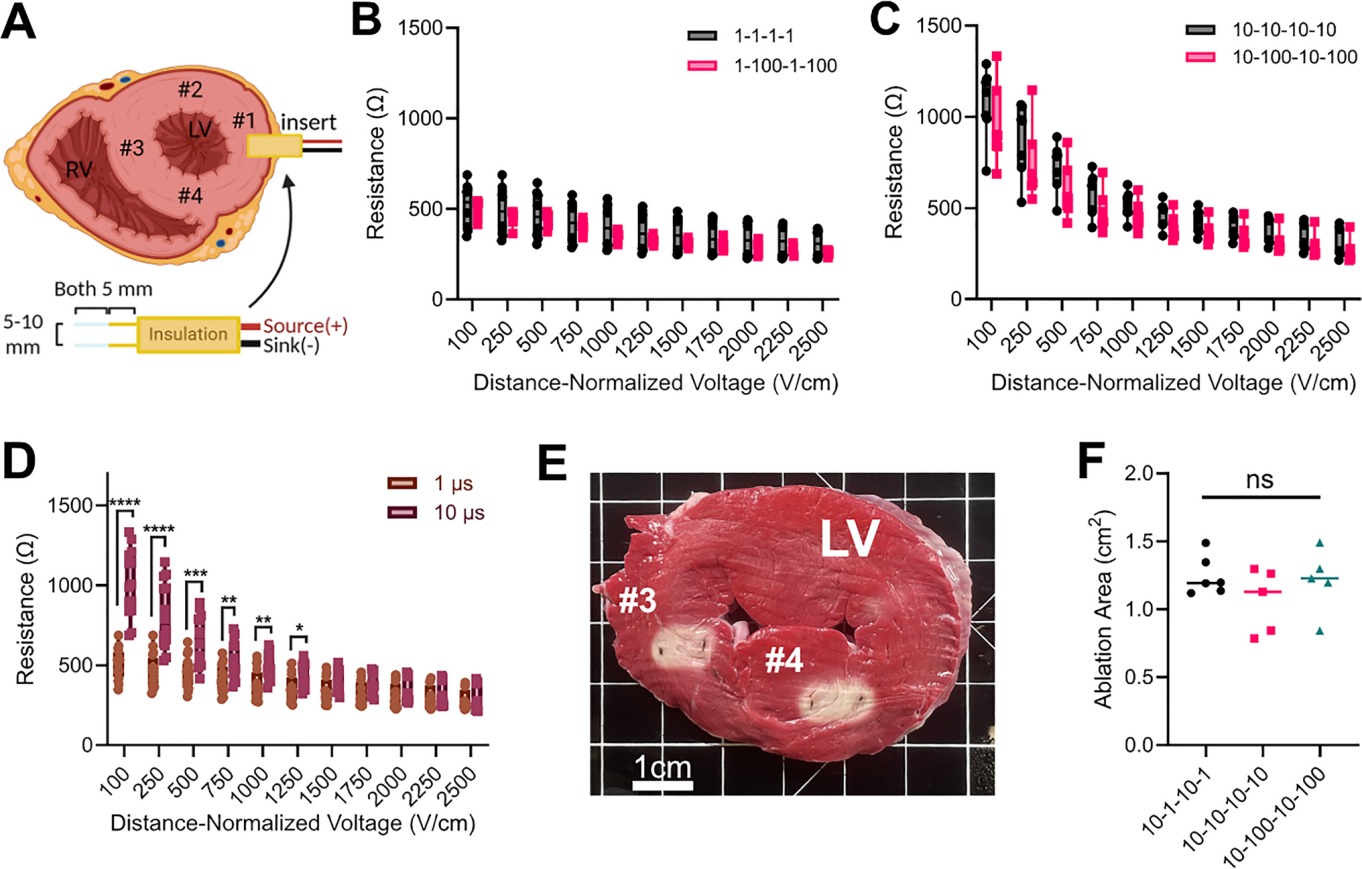
There is no significant difference when varying delays in ex vivo cardiac tissue. **A** Two fixed-spacing 5-mm exposure needle electrodes were inserted 10 mm into fresh porcine heart at 4 locations (#1–4) within the left ventricle (LV). Measured resistance for the **B** 1 µs pulse width and **C** 10 µs pulse width. **D** Resistances for 1 and 10 µs at different distance-normalized voltages. **E** Metabolic 2,3,5-Triphenyl tetrazolium chloride (TTC) staining of cardiac tissue slices ~ 4 hours after treatment with 10 µs pulsed field ablation; red indicates metabolically active tissue. #3 is a 10-1-10-1 treatment and #4 is 10–100-10–100 treatment. **F** Measured lesion areas for various to 10 µs pulsed field ablation treatments. (Panels B-D: *n* = 8; Panel F: *n* ≥ 5; *p < 0.05, **p < 0.01, ***p < 0.001, ****p < 0.0001)

Lastly, we corroborated our findings using ex vivo cardiac tissue (Fig. 4A). We found that the trends persisted with no significant differences in measured current or resistance between delays for both 1 µs and 10 µs pulse widths (Fig. 4A, B). Further, the trends for convergence of the measured resistance as the applied distance-normalized voltage increases persisted (Fig. 4C). There was a significant difference in measured resistances between 1 µs and 10 µs pulse widths from 100 V/cm to 1500 V/cm. From 1750 V/cm to 2500 V/cm, there was no significant difference in either current or resistance between 1 µs and 10 µs pulse widths.

### Waveforms with Comparable Frequency Distributions can have Significantly Different Currents and Resistances

To determine whether the frequency spectrum of the waveform correlated to the measured resistance or applied current, we chose two sets of reciprocal waveforms with comparable impedance distributions and with the same characteristic frequency. The 1-10-1-10 and 10-1-10-1 waveforms have the same characteristic frequency ~ 45 kHz (Supplemental Fig. 4A), but the current (Supplemental Fig. 4B) and resistance (Supplemental Fig. 4C) were significantly different at 50 V/cm, 100 V/cm, 250 V/cm. Similarly, the 2-5-2-5 and 5-2-5-2 waveforms have the same characteristic frequency ~ 71 kHz (Supplemental Fig. 4D), but the current (Supplemental Fig. 4E) and resistance (Supplemental Fig. 4F) are significantly different at 50 V/cm.

### Tissue Ablation Areas are Not Affected by Interphase and Interpulse Delays

**Fig. 5:**
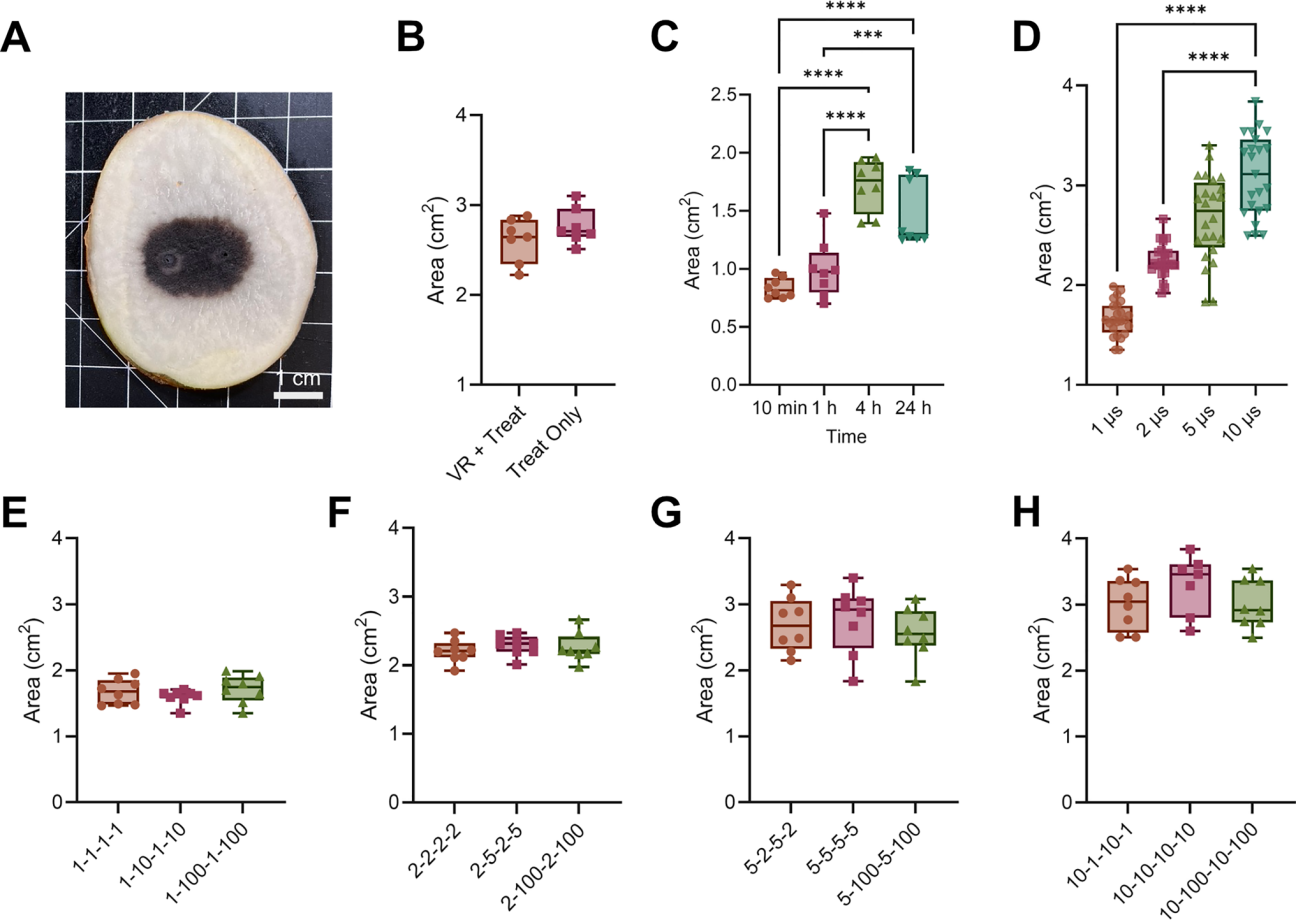
Tissue ablation areas are not affected by interphase and interpulse delays. **A** Representative image of ablation demarcated with brown melanin formation. **B** Ablation areas after either voltage ramp and treatment or treatment alone show no significant difference. **B** Measured ablation areas 10 minutes, 1 hour, 4 hours, and 24 hours after treatment. **C** Ablation areas for each applied pulse width. Measured ablation areas for different delays with **A** 1 µs, **B** 2 µs, **C** 5 µs, and **D** 10 µs pulse width H- FIRE waveforms. **B**, **C**, and **E**–**H**: *n* = 8; **D**: *n* = 24; ***p < 0.001, ****p < 0.0001)

Cell damage in potato tissue is visible due to a natural oxidation process of released intracellular enzymes (polyphenol oxidase) and, subsequent, formation of brown-black melanins (Fig. 5A) [58]. Though cell death may occur immediately following IRE, melanin typically forms within a few hours, allowing for measurement of the ablation at 24 hours after treatment [58, 59]. However, H-FIRE is indicated to possibly produce delayed apoptotic cell death [45]. Therefore, we measured ablations at 10 min, 1 h, 4 h, and 24 h after treatment (Fig. 5C) to determine optimal times for measuring ablations.

We found that ablations were saturated by 24 h; thus, we measured all the ablation areas at 24 h after treatment. Tetrazolium chloride, a redox indicator used to stain for metabolically active and live tissue, was used previously with IRE to validate melanin measurements (Supplemental Fig. 5A) [59]. Here, we verified that the melanin formation at 24 h following H-FIRE matched the area of tissue not stained with tetrazolium chloride (Supplement Fig. 5B, C), indicating that the melanin fully developed within the dead tissue by the time of measurements. This allows for imaging of ablations formed by electric fields impacted by both non-linear electroporation-dependent and thermal effects, not typically replicated *in vitro.*

Since we applied a voltage ramp directly before treatment, we also verified that it did not significantly affect the measured ablations from the treatment with 100 bursts. We found no significant difference in measured ablations between potatoes that received a voltage ramp prior to treatment and potatoes that received treatment only (Fig. 5B).

We see that the ablation area increases as the applied pulse width increases. There are significant differences in ablation area between 1 µs and 10 µs and between 2 µs and 10 µs (Fig. 5D). Within each pulse width, when the delays are varied, there is no significant difference for 1 µs (Fig. 5E), 2 µs (Fig. 5F), 5 µs (Fig. 5G), and 10 µs (Fig. 5H) pulse widths.

Likewise, we measured the ablation areas for 10 µs waveform delivered into the left atrium (Fig. 4E). There were no significant differences in ablation area between the 1 µs, 10 µs, and 100 µs delays (Fig. 4F).

### Local Tissue Conductivity is Not Affected by Interphase or Interpulse Delay

The measured resistance using the two monopolar NanoKnife^®^ probes is dependent on the non-linear electric field distribution and the electroporated tissue volume [60]. We sought to isolate the influence that pulse width and delays had on the local conductivity within the tissue. Using parallel flat plates electrodes, we can deliver a uniform electric field through a cylindrical tissue sample, irrespective of non-linear changes within the tissue [55]. The tissue conductivity at different applied electric fields can be calculated from the measured resistance using the shape factor for a cylinder [52].

**Fig. 6:**
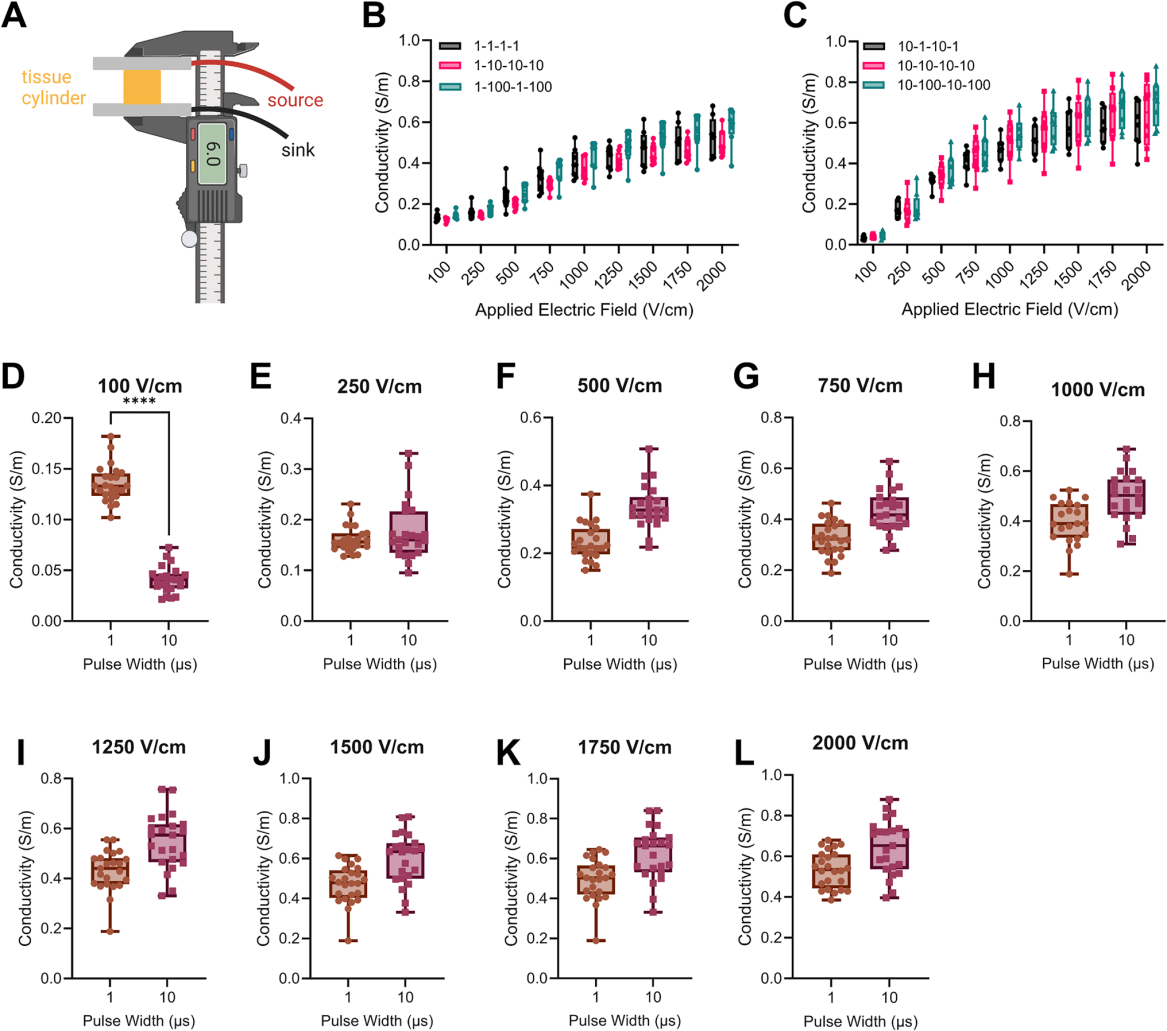
There is not a significant difference in local tissue conductivity between different delays. **A** Cylindrical tissue slices were placed between parallel flat plate electrodes to deliver a uniform electric field. Measured tissue conductivity for the **B** 1 µs pulse width at different delays and **C** 10 µs pulse width at different delays. **D** There is a significant difference in measured conductivity at 100 V/cm. **E**–**L** There is no significant difference in measured conductivity from 250 to 2000 V/cm applied. (Panels B–C, *n* = 8; Panels D–L, *n* = 24; ****p < 0.0001)

The local tissue conductivity using the flat plates exhibited similar trends to the resistance measurements obtained using the monopolar NanoKnife^®^ probes. For both the 1 µs (Fig. 6A) and 10 µs (Fig. 6B) pulse widths, there were no significant differences in tissue conductivity between the delay groups for any applied electric field. The mean bulk conductivities (non-electroporated conductivity) were 0.13 S/m and 0.04 S/m for the 1 µs and 10 µs pulse widths, respectively, with 1 µs pulse width being significantly higher than the 10 µs pulse width at 100 V/cm (Fig. 6C). There were no significant differences between 1 µs and 10 µs for any of the other applied electric fields (Fig. 6D). The median electroporated conductivity plateaued at 0.55 S/m and 0.67 S/m for 1 µs and 10 µs, respectively, with values ranging from 0.40 S/m to 0.85 S/m (Fig. 6E). Further, while the 1 µs pulse width didn’t significantly increase from baseline (100 V/cm) until 750 V/cm (Fig. 6A), the conductivity for 10 µs pulse width significantly increased after 250 V/cm (Fig. 6B).

## Discussion

Our data are the first to experimentally demonstrate that H-FIRE interphase and interpulse delays do not significantly impact the applied current, the tissue resistance, or the local tissue conductivity at a given pulse width and applied voltage. Further, our findings support previous *in vitro* data that delays do not significantly affect ablation areas [49, 61].

Large amounts of fresh mammalian tissue (e.g., hearts) can be difficult to obtain for fundamental studies, tuber tissue provides an essential and high-throughput bench top model, as plant cells are perforated due to an applied electric field and exhibit electroporation-dependent conductivity changes like animal cells [58]. Previous reports have found that potato tissue is permeabilized with conventional 100 µs pulses at electric fields ranging from 200 V/cm to 500 V/cm, with the onset of irreversible electroporation occurring above 500 V/cm [62, 63]. We saw a significant increase in tissue conductivity following 10 µs pulses at 250 V/cm and significant increases in conductivity following 1 µs pulses occurred at 750 V/cm. The increase in ablation areas with pulse width follows previous trends and is generally attributed to the decrease in membrane charging with lower pulse widths and subsequent decrease in pore formation [44, 46, 64]. Our data also support previous *in vitro* literature indicating that electroporation thresholds are not significantly affected by interphase and interpulse delays [49]. The results showing that there are no significant differences in resistance between delays follow this observation; if the conductivity and resistance were different between delays but the lethal thresholds were the same, then we would expect to see differences in ablation areas due to the difference in electric field distribution.

The range of previously characterized bulk conductivities for conventional 100 µs monopolar pulses in potatoes was between 0.03 and 0.05 S/m [62, 63, 65–67]. The mean bulk conductivity for 10 µs pulse widths fell within this range, despite the pulse width being an order of magnitude shorter. This could be attributed to the 10 µs pulse width being roughly 10x the length of the time constant for cell membrane charging (~ 0.7–1.4 µs) of tuber tissue to a square pulse [68, 69].

We found no significant differences in resistance, current, conductivity, or areas between different pulse widths or delays after inducing electroporation. Previously, the conductivity of potatoes treated with conventional IRE at 1000 V/cm was found to be 0.38 S/m [62]. This value falls within the range of conductivities found here for both 1 µs (0.19–0.52 S/m) and 10 µs (0.30–0.68 S/m) at 1000 V/cm. This provides evidence that the electroporated conductivity of tissue is not dependent on the applied waveform. Experimental data and molecular simulations suggest that cell membrane conductivity increases orders of magnitude after pore formation [23, 70–72], so differences in pore density or size induced by different pulse widths may not lead to appreciable differences in the local conductivity of electroporated tissue. Further, since the ablation areas, the non-electroporated tissue conductivity, and electroporated tissue conductivity are not affected by delays, the electric field distribution should be consistent for different delays.

While not explicitly measured here, it is unlikely that interphase or interpulse delays would significantly impact the temperature distribution during PFA. The maximum delay (100 μs) is orders of magnitude lower than the time scale for temperature change due to thermal diffusion in potatoes [73] and mammalian tissue [74]. Indeed, previous experiments have reported temperature rise and decrease on the order of seconds to minutes, both with perfusion [75, 76] and without perfusion [77]. Decreasing the rate of applied bursts can significantly affect the temperature generated due to this timescale [48]. Further, if there were temperature differences between delays, then we would see evidence through significant differences in temperature-dependent values, such as current, resistance, local conductivity, and ablations.

This study demonstrates that H-FIRE interphase and interpulse delays do not significantly impact applied current, tissue resistance, ablation areas, or local tissue conductivity. These findings enhance our understanding of the mechanisms underlying pulsed field ablation (PFA) and suggest that H-FIRE treatment parameters can be optimized with greater flexibility in delay durations without compromising treatment efficacy. Clinically, these results are particularly relevant for cardiac PFA applications, where treatment protocols must prioritize safety, precision, and efficiency. The lack of significant impact from delays simplifies waveform parameterization, potentially reducing procedural complexity and facilitating the development of standardized protocols for catheter-based ablations.

A key limitation of this study is the use of fresh ex vivo cardiac tissue rather than in vivo porcine models. However, previous work in liver [78] and a recent in vivo cardiac characterization study [79] support that ex vivo tissue properties are representative of in vivo values and ablations. By elucidating how delays affect treatment efficacy, this work provides a foundation for reducing the parameterization burden in future in vivo studies. This shift would allow researchers to focus on parameters directly influencing clinical outcomes, thereby increasing the statistical power of preclinical and clinical trials. Future studies should validate these findings in vivo and explore their integration into clinical workflows, paving the way for safer and more effective H-FIRE treatments in cardiac and other applications. Further, while we found similar ablation and resistance trends for potato and cardiac tissue, future work should expand the cardiac data to cover all the conditions explored within the tuber tissue and evaluate data within malignant tissue that may possess higher patient-to-patient variability. Further, while we did not observe a difference between the delays that span three orders of magnitude, we expect that electroporation effects would diminish at some higher delay outside of the range of our electroporation generator. Future experiments should quantify the bounds in which the delays do not affect the electroporation outcomes.

### Clinical Perspectives

PFA parameters used within the clinic are not often disclosed, providing little information about how H-FIRE parameterization affects outcomes. This produces unnecessary preclinical and clinical evaluations to repeat data possibly already gathered. Our work explored the influence of H-FIRE waveform delays on tissue resistance, ablation areas, and local conductivity in both cardiac and tuber tissue models. Our findings have direct implications for H-FIRE parameterization and study group designs in future, as experimental arms may not need to explore delays but focus on parameters that significantly affect clinical outcomes. Further, the research presented aligns with our broader efforts, including ongoing collaborations with the FDA, to establish standardized treatment protocols for cardiac PFA.

## Supplementary Information

Below is the link to the electronic supplementary material.

Supplementary file1 (PDF 578 kb)

## Data Availability

The data that support the findings of this study are available within the article and its supplementary material. Further requests can be made to the corresponding author.

## Abbreviations

PFA: Pulsed field ablation
IRE: Irreversible electroporation
H-FIRE: High-frequency irreversible electroporation
EFT: Electric field threshold
