# Supplementary material for: Effects of Interphase and Interpulse Delays on Tissue Impedance and Pulsed Field Ablation
Source: Ann Biomed Eng. 2025 May 16;53(8):1962–74. doi: 10.1007/s10439-025-03757-4 (PMC12283778; doi:10.1007/s10439-025-03757-4)
Supplement: Supplementary file 1 — Supplementary file1 (PDF 578 kb) [file 10439_2025_3757_MOESM1_ESM.pdf]

Supplement

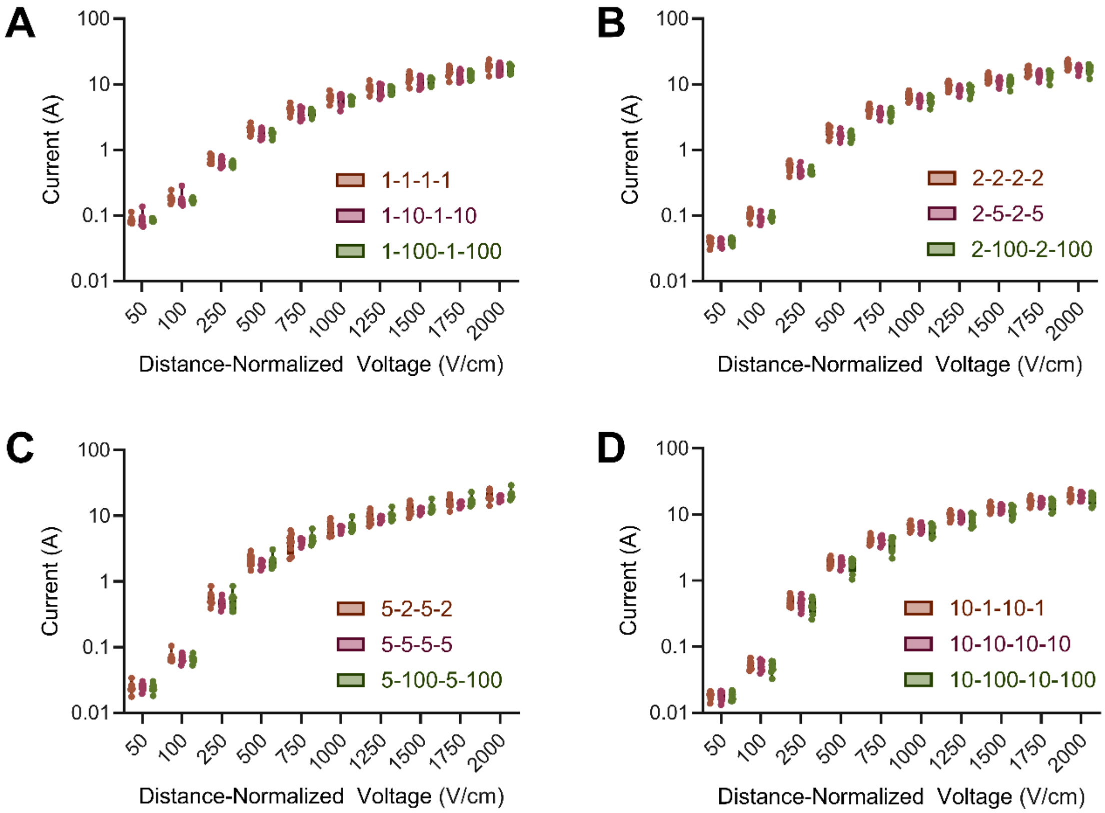

Supplemental Figure 1. There is not a significant difference in current applied to the tissue when varying delays. Current was measured using 2 monopolar probes with a 1.0 cm center-to-center spacing. Applied current measurement from 50 V/cm to 2000 V/cm for **A)** 1 µs, **(B)** 2 µs, **(C)** 5 µs, and **(D)** 10 µs pulse width H-FIRE waveforms with delays varying from 1 µs to 100 µs. (n = 8)

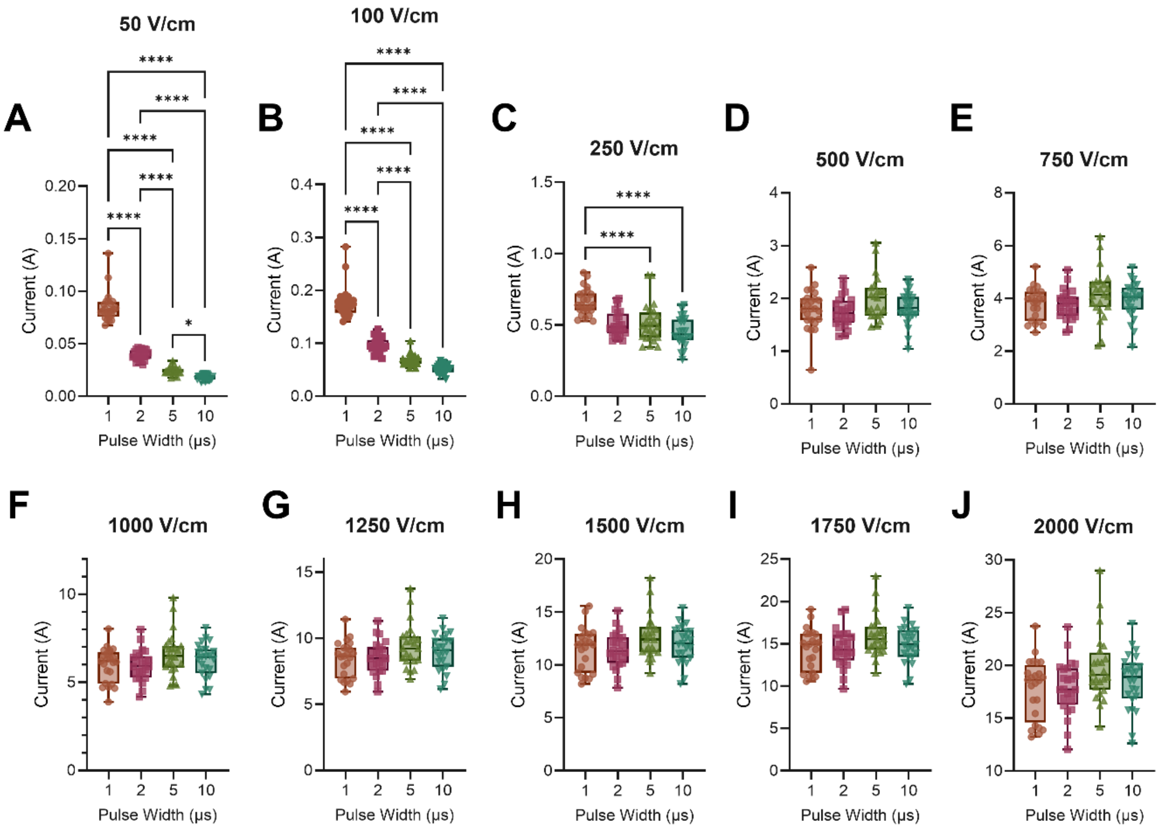

Supplemental Figure 2. A) At sub-electroporation thresholds, there is a significant difference for current between pulse widths, with higher pulse widths having lower applied currents. **B)** At 100 V/cm, there is still a significant difference between different pulse widths, but the applied current significantly increases for each pulse width **C)** At 250 V/cm, there is only a significant difference in current between the 1 μs and 10 μs and between 1 μs and 5 μs. **D-J)** At high applied electric fields, there is not a significant difference in resistance between pulse widths. ($n = 24$, * $p < 0.05$, ** $p < 0.01$, *** $p < 0.001$, **** $p < 0.0001$).

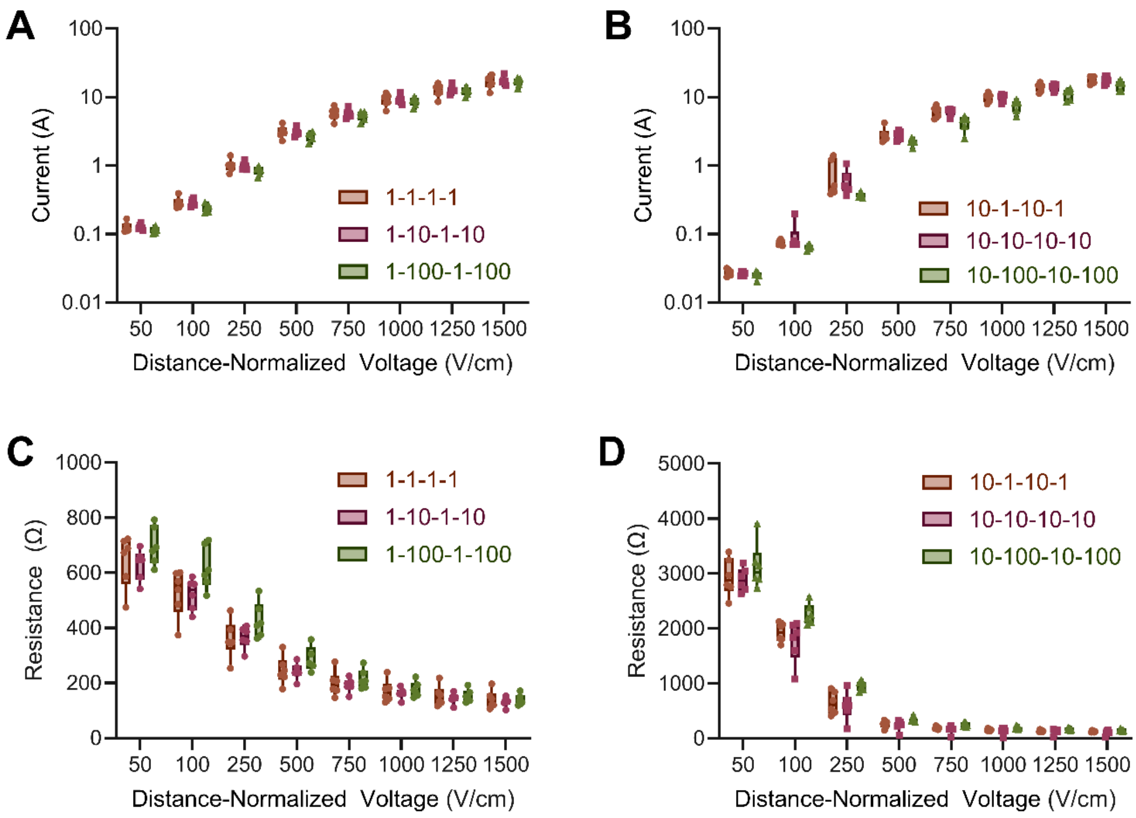

Supplemental Figure 3. There is not a significant difference in current and resistance between delays with other treatment geometries. Current and resistance were measured using 2 monopolar probes with a 1.5 cm center-to-center spacing. There is no significant difference in measured current between delays for **A)** 1 µs and **B)** 10 µs pulse widths. There is no significant difference in measured resistance between delays for **A)** 1 µs and **B)** 10 µs pulse widths. (n = 8)

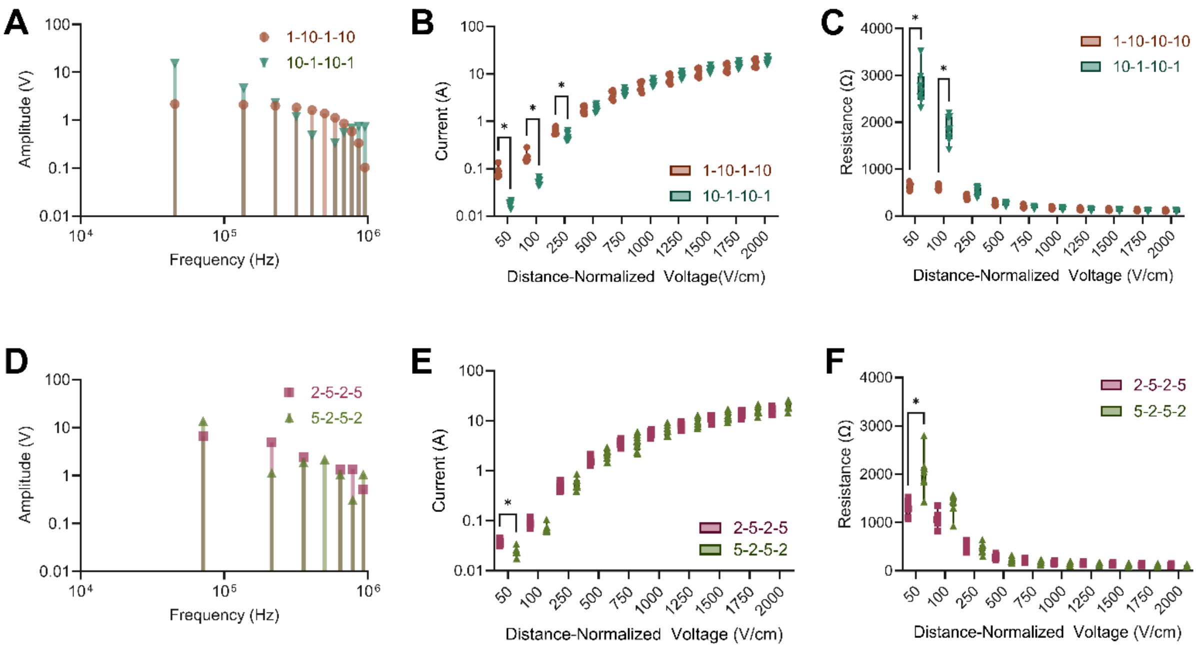

Supplemental Figure 4. Waveforms with comparable frequency distributions can have significantly different currents and resistances. A) Frequency distribution for the 1-10-1-10 and 10-1-10-1 waveforms. **B)** Current and **C)** Resistance significantly differ at 50, 100, and 250 V/cm. **D)** Frequency distributions for the 2-5-2-5 and 5-2-5-2 waveforms. Current and resistance significantly differ at 50 V/cm. (n=8; * $p < 0.05$).

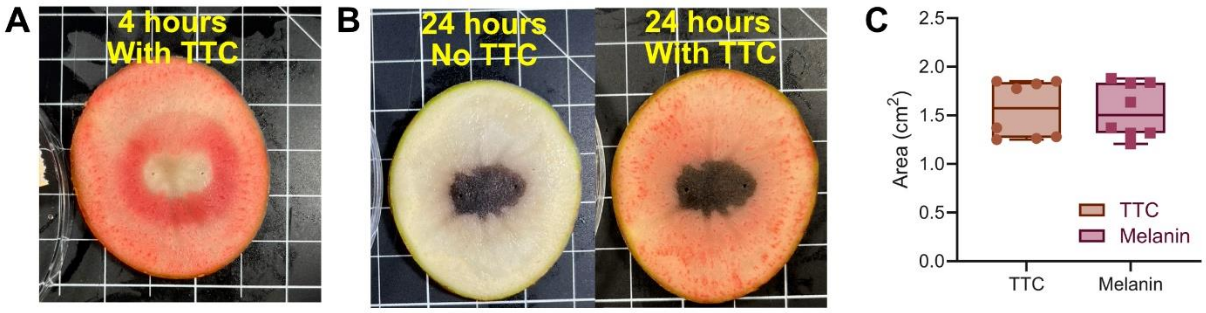

Supplemental Figure 5. Comparison of measured ablation areas using tetrazolium chloride (TTC) or melanin staining. Potatoes were treated with 1500 V/cm of 5-5-5-5. At 10 minutes, 1 hour, 4 hours, and 24 hours post-treatment ablations were measured either using the melanin generated by oxidation or through staining using tetrazolium chloride (TTC). Melanin did not form in the 10-minute, 1-hour, or 4-hour group, but **A)** TTC did allow for visualization of ablations in the 10-minute, 1-hour, and 4-hour groups. **B)** At 24 hours the melanin formed and the region of melanin and TTC stain were equal, but there was **C)** there was no significant difference between the 4-hour TTC group areas and the 24-hour melanin areas, indicating that melanin formed within the ablation that was already present but not visible without viability staining. (n = 8)
